# Posterior Reversible Encephalopathy Syndrome Following Massive Blood Transfusion in a Case of Pelvic Schwannoma

**DOI:** 10.1155/cria/4446356

**Published:** 2026-08-26

**Authors:** Jagdeep Sharma, Harsimran Walia, Arvind Guru, Lalita Gouri Mitra, Rahat Brar, Ashish Gulia

**Affiliations:** ^1^ Department of Anesthesia, Critical Care & Pain, HBCH & RC Mullanpur, New Chandigarh, Punjab, India; ^2^ Homi Bhabha National Institute, Mumbai, India, hbni.ac.in; ^3^ Department of Surgical Oncology, HBCH & RC Mullanpur, New Chandigarh, Punjab, India; ^4^ Department of Radiology, HBCH & RC Mullanpur, New Chandigarh, Punjab, India

**Keywords:** general anesthesia, massive blood transfusion, posterior reversible encephalopathy syndrome

## Abstract

Posterior reversible encephalopathy syndrome (PRES) in the postoperative period poses diagnostic and management challenges. Large pelvic soft‐tissue tumors can cause substantial blood loss, requiring massive blood transfusion (MBT). We present a case of a 46‐year‐old male who underwent excision of a large pelvic ancient schwannoma, complicated by intraoperative massive blood loss of 8 L, necessitating transfusion of 9 units of packed red blood cells. The patient subsequently developed PRES in the postoperative period, as confirmed by MRI. The diagnosis and management of rare perioperative neurological complications such as PRES remain challenging. They require a high index of suspicion, vigilant clinical evaluation, prompt neuroimaging, and coordinated multidisciplinary care to enhance patient safety and outcomes.

## 1. Introduction

Pelvic soft‐tissue tumors are a diverse group of benign and malignant mesenchymal tumors arising from muscle, fibrous tissue, fat, blood vessels, and peripheral nerves within the pelvic cavity. Their deep‐seated location, involvement of critical neurovascular structures, and nonspecific clinical features often lead to delayed diagnosis and complex management [1]. Schwannomas are benign peripheral nerve sheath tumors, and an ancient schwannoma is a rare variant characterized by significant degenerative changes [2]. Sacral nerve root schwannomas most commonly arise from the S1–S3 nerve roots, although S4–S5 involvement has also been reported. Wide, complete surgical resection is the cornerstone of treatment and is associated with excellent patient outcomes. Large tumors (> 10 cm), particularly those with difficult access because of their deep‐seated location and proximity to major vessels, increase the risk of severe intraoperative bleeding [3].

The postoperative period after these supramajor surgeries is marked by complex physiological changes, making the diagnosis and management of rare neurological disorders extremely challenging. Massive blood transfusion (MBT) carries risks of complications, including coagulopathy, hypocalcemia, hyperkalemia, transfusion‐related lung injury, transfusion‐related cardiac overload, and hypothermia [4]. We report this very rare case of a pelvic ancient schwannoma that developed posterior reversible encephalopathy syndrome (PRES) in the postoperative period after intraoperative MBT.

## 2. Case Report

A 46‐year‐old male patient (height 167 cm; weight 70 kg; body mass index 25.1) with 4 months of abdominal pain, no known medical comorbidities, and no history of drug or food allergies was scheduled for elective excision of a pelvic mass. Preoperative radiologic evaluation revealed a large pelvic soft‐tissue mass measuring 13 × 6 × 6 cm. In the OR, the patient was connected to a multichannel monitor (Phillips) and monitored for noninvasive arterial blood pressure, electrocardiogram (leads II and V5), continuous pulse oximetry (SpO_2_), respiratory rate, and end‐tidal carbon dioxide. A radial arterial line was placed for continuous invasive blood pressure monitoring, and pulse pressure variation (PPV) was used for advanced hemodynamic monitoring given anticipated massive blood loss. Two large‐bore 16‐G intravenous cannulas and one 18‐G cannula were secured to enable rapid fluid resuscitation and administration of blood products.

All baseline parameters were recorded; blood pressure at induction was 140/84 mmHg, and standard monitoring was continued throughout the intraoperative period. For this procedure, preoperative antibiotic prophylaxis was administered with piperacillin–tazobactam (4000/500 mg). A crystalloid infusion of Ringer’s lactate was started at 2 mL/kg/h via a fluid warmer. A thoracic epidural was inserted at T9‐10, and after adequate preoxygenation, general anesthesia was induced per protocol with propofol (2 mg/kg) and fentanyl (2 mcg/kg). Endotracheal intubation was facilitated after muscle relaxation with the nondepolarizing muscle relaxant vecuronium (0.1 mg/kg). Subsequently, anesthesia was maintained with 2.5% sevoflurane in an oxygen–air mixture throughout the procedure.

Intraoperatively, estimated blood loss was 8 L over 6 h, with multiple episodes of severe anemia. The lowest recorded hemoglobin level was 5.5 gm/dL, necessitating rapid blood transfusion. Volume resuscitation was managed with 3 L of Ringer’s lactate, 1 L of colloid, 9 units of packed red blood cells (PRBCs), 8 units of random donor platelets (RDPs), and 6 units of fresh frozen plasma (FFP). The patient remained relatively hemodynamically stable, with mean arterial pressure (MAP) ranging from 45 to 94 mmHg. Intraoperative hypotension was managed with intermittent mephentermine boluses of 6 mg (total cumulative dose of 24 mg) and a transient norepinephrine infusion (0.1–0.2 mcg/kg/min). The total duration of surgery and anesthesia was approximately 8 h. The patient was hemodynamically stable and normothermic, and arterial blood gas analysis (Table 1a) was within the normal range. The patient was conscious, obeying commands, and extubated after administration of neostigmine reversal (2.5 mg) and glycopyrrolate (0.4 mg).

**TABLE 1:** Arterial blood gas analysis.

Parameters	1a (end surgery)	1b (PACU)	1c (POD‐3)	Reference range
pH	7.38	7.28	7.42	(7.35–7.45)
PaO_2_ (mmHg)	124	139	144	(83.0–100)
PaCO_2_ (mmHg)	41	45	32.7	(35–45)
Sodium (mmol/L)	137	138	141	(135–145)
Potassium (mmol/L)	4.0	4.2	3.6	(3.5–5.5)
Calcium (mmol/L)	1.08	1.14	1.05	(1.15–1.29)
Lactates (mmol/L)	1.2	3.3	0.8	(0.5–1.5)
Anion gap (with K^+^) (mmol/L)	10.6	13	11	
Bicarbonates (mmol/L)	24.2	21	22	
Hemoglobin (g/dL)	9.6	8.9	7.8	(12.0–16.5 gm/dL)

There were no hypertensive surges in the postoperative period, with vital signs including a heart rate of 88/min, a blood pressure of 130/84 mmHg, and an oxygen saturation of 97%. Within an hour in the PACU, the patient became unconscious, with hemodynamic parameters remaining stable. The airway was secured, and the patient was transferred to the intensive care unit. ABG was unremarkable, with normal ionized calcium levels and no evidence of respiratory failure or other metabolic abnormality, except for a lactate of 3.3 mmol/L (Table 1b). A chest radiograph showed clear lung fields.

**FIGURE 1:**
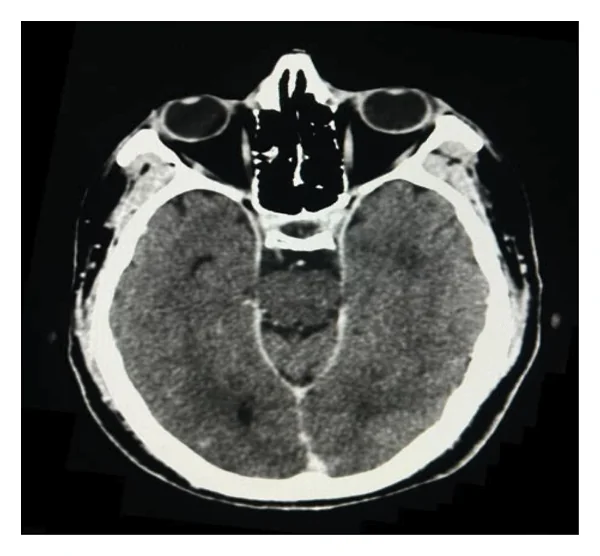
Noncontrast axial brain CT at the level of the orbits and posterior fossa, obtained on POD‐1. No acute hemorrhage, mass effect, midline shift, or hydrocephalus is observed. No definite hypodensity is identified in the visualized occipital lobes at this stage.

On POD‐1, the patient regained consciousness but experienced a generalized tonic–clonic seizure (GTCS) lasting 2–3 minutes. The GTCS was managed with inj. lorazepam 2 mg and inj. levetiracetam 1 g. Given the suspicion of spinal cord manipulation during tumor resection, inj. vancomycin 1 g BD was started empirically. Cerebrospinal fluid (CSF) analysis, including biochemistry and culture, was performed to rule out infectious neurological complications (CSF cell count: 18 cells/μL; glucose: 68 mg/dL; protein: 58 mg/dL; no organisms). A contrast‐enhanced computed tomography (CECT) scan was also performed to rule out hemorrhage, and it was normal. Figure 1

**FIGURE 2:**
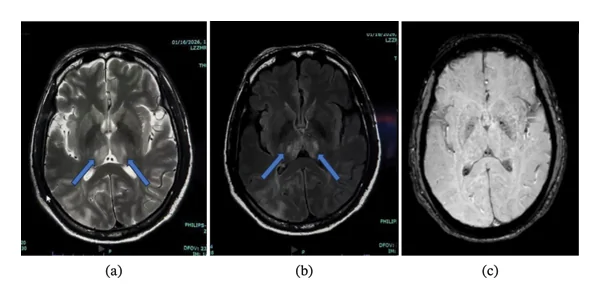
Axial MR images of the brain at the level of the thalami: (a) T2‐weighted image and (b) corresponding FLAIR image showing symmetric hyperintense signal in the bilateral thalami (blue arrows), reflecting the central/deep gray matter variant of PRES. (c) Corresponding axial SWI at the same level showing no blooming to suggest acute hemorrhage or microhemorrhage within the thalamic lesions.

**FIGURE 3:**
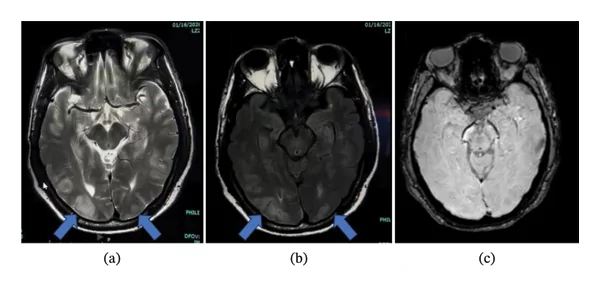
Axial MR images of the brain at the level of the midbrain: (a) T2‐weighted image and (b) corresponding FLAIR image showing hyperintense signal in the cortical and subcortical white matter of the bilateral occipital lobes, more conspicuous on FLAIR due to CSF signal suppression. (c) Susceptibility‐weighted imaging (SWI) demonstrating no evidence of acute hemorrhage.

On POD‐3, the patient was conscious, well‐oriented, hemodynamically stable, and seizure‐free for 48 h. ABG was within normal limits (Table 1c), after which the patient was extubated. After sterile cultures and negative microbiological investigations, with no clinical evidence of infection, vancomycin was discontinued after 48–72 h. After 10 h, the patient was reintubated due to another episode of loss of consciousness. Brain magnetic resonance imaging (MRI) with contrast was performed on POD‐4, which showed evidence of PRES (Figures 2 and 3).

Following this, we continued neuroprotective measures, and the patient underwent tracheostomy and was gradually weaned from the ventilator. The patient was fully conscious, hemodynamically stable, and seizure‐free and was transferred to the ward on POD‐9. He was discharged home on POD‐14 on maintenance antiepileptic therapy. At one‐week follow‐up, the patient was conscious and oriented, with no neurological deficits, and was decannulated. At six months of regular follow‐ups, the patient was doing well, seizure‐free, with complete neurological recovery (see Table 2).

**TABLE 2:** Event timeline.

Event	Timings
1. Surgery completed	0 h
2. Loss of consciousness	1 h postop
3. First seizure episode	POD‐1
4. MRI‐confirmed diagnosis of PRES	POD‐4
5. Ward transfer	POD‐9
6. Discharge home	POD‐14
7. Decannulation	At the one‐week follow‐up

## 3. Discussion

PRES is a rare neurological disorder with an age‐ and sex‐adjusted incidence of approximately 2.7–3.1 cases per 100,000 people per year. As a rare and likely underdiagnosed complication, the exact incidence of PRES in the postoperative period remains unclear. PRES following MBT is also exceedingly uncommon. It typically presents with severe headache, altered mentation or stupor, drowsiness, visual disturbances, and seizures that may be focal or generalized tonic–clonic. Common causes include acute hypertension, preeclampsia, eclampsia, immunosuppressive therapy, acute kidney injury, and sepsis [5].

Our patient underwent an extremely challenging intraoperative tumor resection; this extensive resection was associated with episodes of severe hypotension, managed effectively with sympathomimetic drugs, without extreme hypertensive surges. There were no major fluctuations in blood pressure in the postoperative period. He developed stupor in the early postoperative phase, within 6 h of MBT, followed by an episode of GTCS after 12 h. However, on presentation, there were no associated MBT complications, including hypocalcemia, dilutional coagulopathy, hypothermia, or volume overload. The patient also displayed an atypical, waxing‐and‐waning level of consciousness, cycling between complete wakefulness and unresponsiveness, making diagnosis extremely difficult.

MBT‐induced PRES is exceptionally rare. Multiple blood transfusions can trigger acute hyperperfusion, hyperviscosity after rapid transfusion, endothelial dysfunction, cytokine‐mediated vascular injury, and fluid leakage, which primarily manifest as vasogenic edema in the posterior regions of the brain (especially the occipital lobe and thalamus). The posterior cerebral circulation has a lower density of sympathetic nerve fibers, reducing the vessels’ ability to constrict to protect the capillary beds and increasing vulnerability to injury. Previous studies have also highlighted the development of PRES in patients receiving multiple blood transfusions for chronic severe anemia or other causes of bleeding [6].

Noncontrast CT sections of the brain showed no evidence of acute intracranial hemorrhage, mass effect, midline shift, or hydrocephalus. The visualized brain parenchyma showed preserved gray‐white matter differentiation. Because CT is less sensitive than MRI for subtle vasogenic edema in PRES, it did not show findings consistent with the occipito‐thalamic changes later demonstrated on MRI—underscoring the need for MRI in cases of high clinical suspicion despite an unremarkable CT.

MRI of the brain, particularly T2‐weighted and FLAIR sequences, is the gold standard for diagnosing PRES, showing hyperintense lesions in the cortical and subcortical white matter. On our patient’s MRI, diffusion‐weighted imaging (DWI) over the FLAIR hyperintense areas in the bilateral occipital and thalamic regions showed no significant hyperintensity to suggest true restricted diffusion, and the corresponding apparent diffusion coefficient (ADC) maps showed facilitated diffusion (elevated ADC signal) in these areas. The lesions predominantly involved the subcortical white matter, with relative sparing of the overlying cortical ribbon. This absence of diffusion restriction, with elevated ADC values, is characteristic of vasogenic edema and helped distinguish PRES from an acute ischemic infarction, which typically shows restricted diffusion (low ADC) [7]. The overall imaging findings on MRI were interpreted by the radiologist as consistent with PRES. The acute management of PRES is primarily supportive, focusing on maintaining hemodynamic parameters; it must be prompt in identifying and controlling triggers, use antiepileptic drugs in the presence of seizures, and be systematic in preventing progression and irreversible neurological injury.

## 4. Conclusion

The diagnosis and management of PRES in the postoperative period are extremely challenging. While MBT carries inherent risks, recognizing rare neurological complications such as PRES requires a high index of suspicion, vigilant clinical evaluation, prompt neuroimaging, and precise management through coordinated, multidisciplinary care to enhance patient safety and optimize outcomes.

## Funding

No funding was received for this manuscript.

## Consent

This case report was published with the patient’s written consent.

## Conflicts of Interest

The authors declare no conflicts of interest.

## Data Availability

The data that support the findings of this study are available on request from the corresponding author. The data are not publicly available due to privacy or ethical restrictions.

## Appendix A

1. We present a case of a 46‐year‐old male who underwent excision of a large pelvic ancient schwannoma, complicated by intraoperative massive blood loss of 8 L, necessitating transfusion of 9 units of packed red blood cells.
2. All baseline parameters were recorded; blood pressure at induction was 140/84 mmHg, and standard monitoring was continued throughout the intraoperative period
3. Intraoperatively, estimated blood loss was 8 L over 6 h, with multiple episodes of severe anemia. The lowest recorded hemoglobin level was 5.5 gm/dL, necessitating rapid blood transfusion. Volume resuscitation was managed with 3 L of Ringer’s lactate, 1 L of colloid, 9 units of packed red blood cells (PRBCs), 8 units of random donor platelets (RDPs), and 6 units of fresh frozen plasma (FFP). The patient remained relatively hemodynamically stable, with mean arterial pressure (MAP) ranging from 45 mmHg to 94 mmHg. Intraoperative hypotension was managed with intermittent mephentermine boluses of 6 mg (total cumulative dose of 24 mg) and a transient norepinephrine infusion (0.1–0.2 mcg/kg/min).
4. There were no hypertensive surges in the postoperative period, with vital signs including a heart rate of 88/min, a blood pressure of 130/84 mmHg, and an oxygen saturation of 97%.
5. Cerebrospinal fluid (CSF) analysis, including biochemistry and culture, was performed to rule out infectious neurological complications (CSF cell count: 18 cells/μL; glucose: 68 mg/dL; protein: 58 mg/dL; no organisms)
6. After sterile cultures and negative microbiological investigations, with no clinical evidence of infection, vancomycin was discontinued after 48–72 h
7. Following this, we continued neuroprotective measures, and the patient underwent tracheostomy and was gradually weaned from the ventilator. The patient was fully conscious, hemodynamically stable, and seizure‐free and was transferred to the ward on POD‐9. He was discharged home on POD‐14 on maintenance antiepileptic therapy. At one‐week follow‐up, the patient was conscious and oriented, with no neurological deficits, and was decannulated. At six months of regular follow‐ups, the patient was doing well, seizure‐free, with complete neurological recovery.
9. Our patient underwent an extremely challenging intraoperative tumor resection; this extensive resection was associated with episodes of severe hypotension, managed effectively with sympathomimetic drugs, without extreme hypertensive surges. There were no major fluctuations in blood pressure in the postoperative period. He developed stupor in the early postoperative phase, within 6 h of MBT, followed by an episode of GTCS after 12 h
10. MBT‐induced PRES is exceptionally rare. Multiple blood transfusions can trigger acute hyperperfusion, hyperviscosity after rapid transfusion, endothelial dysfunction, cytokine‐mediated vascular injury, and fluid leakage, which primarily manifest as vasogenic edema in the posterior regions of the brain (especially the occipital lobe and thalamus). The posterior cerebral circulation has a lower density of sympathetic nerve fibers, reducing the vessels’ ability to constrict to protect the capillary beds and increasing vulnerability to injury. Previous studies have also highlighted the development of PRES in patients receiving multiple blood transfusions for chronic severe anemia or other causes of bleeding [6].
11. Noncontrast CT sections of the brain showed no evidence of acute intracranial hemorrhage, mass effect, midline shift, or hydrocephalus. The visualized brain parenchyma showed preserved gray‐white matter differentiation. Because CT is less sensitive than MRI for subtle vasogenic edema in PRES, it did not show findings consistent with the occipito‐thalamic changes later demonstrated on MRI—underscoring the need for MRI in cases of high clinical suspicion despite an unremarkable CT.
12. MRI of the brain, particularly T2‐weighted and FLAIR sequences, is the gold standard for diagnosing PRES, showing hyperintense lesions in the cortical and subcortical white matter. On our patient’s MRI, diffusion‐weighted imaging (DWI) over the FLAIR hyperintense areas in the bilateral occipital and thalamic regions showed no significant hyperintensity to suggest true restricted diffusion, and the corresponding apparent diffusion coefficient (ADC) maps showed facilitated diffusion (elevated ADC signal) in these areas. The lesions predominantly involved the subcortical white matter, with relative sparing of the overlying cortical ribbon. This absence of diffusion restriction, with elevated ADC values, is characteristic of vasogenic edema and helped distinguish PRES from an acute ischemic infarction, which typically shows restricted diffusion (low ADC) [7]. The overall imaging findings on MRI were interpreted by the radiologist as consistent with PRES.
